# Investigation of Solid *D*_2_ for UCN Sources

**DOI:** 10.6028/jres.110.076

**Published:** 2005-08-01

**Authors:** F. Atchison, K. Bodek, B. van den Brandt, T. Bryś, M. Daum, P. Fierlinger, P. Geltenbort, M. Giersch, P. Hautle, M. Hino, R. Henneck, M. Kasprzak, K. Kirch, J. Kohlbrecher, J. A. Konter, G. Kühne, M. Kuźniak, K. Mishima, A. Pichlmaier, D. Rätz, A. Serebrov, M. Utsuro, A. Wokaun, J. Zmeskal

**Affiliations:** Paul Scherrer Institut, Villigen, Switzerland; Jagellonian University, Cracow, Poland; Paul Scherrer Institut, Villigen, Switzerland; Paul Scherrer Institut, Villigen, Switzerland; ETH, Zürich, Switzerland; Paul Scherrer Institut, Villigen, Switzerland; ILL, Grenoble, France; Austrian Academy of Sciences, Vienna, Austria; Paul Scherrer Institut, Villigen, Switzerland; Research Reactor Inst., Kyoto University, Osaka, Japan; Paul Scherrer Institut, Villigen, Switzerland; Jagellonian University, Cracow, Poland; Paul Scherrer Institut, Villigen, Switzerland; Paul Scherrer Institut, Villigen, Switzerland; Jagellonian University, Cracow, Poland; Research Center for Nuclear Physics, Osaka, Japan; Paul Scherrer Institut, Villigen, Switzerland; Petersburg Nuclear Physics Institute, Gatchina, RussiaPaul Scherrer Institut, Villigen, Switzerland; Research Center for Nuclear Physics, Osaka, JapanResearch Reactor Inst., Kyoto University, Osaka, Japan; Paul Scherrer Institut, Villigen, SwitzerlandETH, Zürich, Switzerland; Austrian Academy of Sciences, Vienna, Austria

**Keywords:** catalyser, cold neutron beam, cryogenic converter, ortho-deuterium, ortho-para conversion, Raman spectroscopy, scattering cross sections, single crystal, solid deuterium, ultracold neutrons

## Abstract

Solid deuterium (sD_2_) will be used for the production of ultra-cold neutrons (UCN) in a new generation of UCN sources. Scattering cross sections of UCN in sD_2_ determine the source yield but until now have not been investigated. We report first results from transmission and scattering experiments with cold, very cold and ultra-cold neutrons on sD_2_ along with light transmission and Raman scattering studies showing the influence of the sD_2_ crystal properties.

## 1. Introduction

Solid deuterium (sD_2_) is of great importance for a whole class of new sources of ultracold neutrons (UCN). Theoretically [1–3] and experimentally [4–6] it was shown that sD_2_ at sufficiently low temperature (around 5K), with high enough purity (less than 0.2 % ordinary hydrogen) and with high ortho concentration (*c*_o_ > 0.98) offers the possibility for ultra-cold neutron sources with about two orders of magnitude higher UCN densities as compared to the present best sources. Several sD_2_ based sources are currently under construction worldwide. Their common principle is to expose sD_2_ to a high flux of cold neutrons in order to produce UCN by down-scattering and to extract the UCN from sD_2_ into vacuum and to guide them to storage volumes and experiments. Source performance obviously depends crucially on a high extraction efficiency of UCN from sD_2_. Efficient extraction allows the equilibrium UCN density to build up faster and, if desired, to deliver a larger continuous UCN flux to experiments. One can expect D_2_ crystal properties to have an influence on the extraction efficiency. Indications for considerable changes in scattering of very cold neutrons exist [7], depending on the procedures used for D_2_ crystal preparation. Crystal properties which could be manipulated and potentially influence the extraction efficiency of UCN are of special interest and are the focus of the present studies.

## 2. The Experimental Setup

Solid D_2_ samples can be frozen from the liquid in a cryogenic cell. The cell is mounted on a ^4^He flow cryostat and allows for neutron transmission and scattering experiments with simultaneous optical access. The sample thickness in the neutron beam direction is 10 mm, the optical path length perpendicular to it is 72 mm. The neutron beam windows are made out of AlMg3 alloy machined to 150 µm thickness, the cold optical windows are 3 mm thick sapphire. The thermal shield (80 K to 100 K) surrounds the target cell but leaves the optical access open. Outside of the vacuum, the two optical windows are equipped for optical photography and Raman spectroscopy, respectively. The sample can be easily cooled to 5K and by pumping on the He to below 3 K. A dedicated D_2_ gas system is used for purification and para-to-ortho conversion. Ortho-D_2_ is produced at temperatures around the triple point using OXISORB^®^^2^ [8,9] as a catalyst. The target cryostat and cell, the gas system and the optical systems are described in detail in [10]. Three neutron transmission and scattering setups have been used at the Paul Scherrer Institut, Villigen, Switzerland (cold neutrons, CN, SANS-I instrument [11]) and at the Institute Laue-Langevin, Grenoble, France (very cold, VCN, and UCN, PF2 instrument [12]), always using essentially the same strategy: preparation of the respective neutron beam using a velocity selector (PSI) or a chopper (ILL) in connection with adequate collimation; detection of transmitted and, in part, of scattered neutrons in 2D-detectors.

## 3. Raman Spectroscopy, Light and Neutron Transmission

**Fig. 1:**
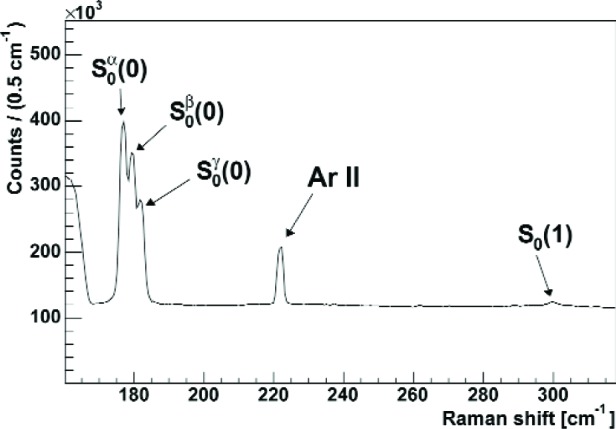
Rotational Raman spectrum of solid ortho-D_2_ (98.6 %).

Rotational Raman spectroscopy is done for two major reasons (see Fig. 1): a) it allows direct monitoring of the ortho-D_2_ concentration of the sample by measuring the intensity ratio of the lines belonging to ortho- [S_0_(0)] and para-D_2_ [S_0_(1)]; b) it yields information about the (hcp-) crystallite orientation in the sample by measuring the intensity distribution between the multiplet lines *α*, *β*, and *γ* which belong to the angular momentum substrates *m* = ±1, ±2, and 0, respectively, of the *J* = 2 final state of the S_0_(0) transition [13]. Earlier, we used vibrational Raman spectroscopy for the investigation of gaseous D_2_ samples at 300 K [14], however, with only *J* = 0 and *J* = 1 states populated at low temperature, the purely rotational transitions yield more information.

**Fig. 2:**
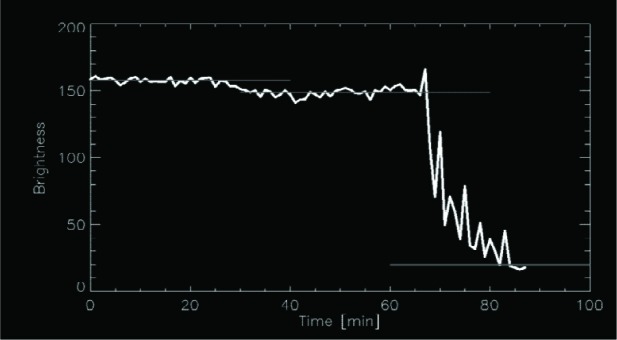
The optical transparency of sD_2_ as deduced from the analysis of image brightness over a period of thermal cycling of the sample.

**Fig. 3:**
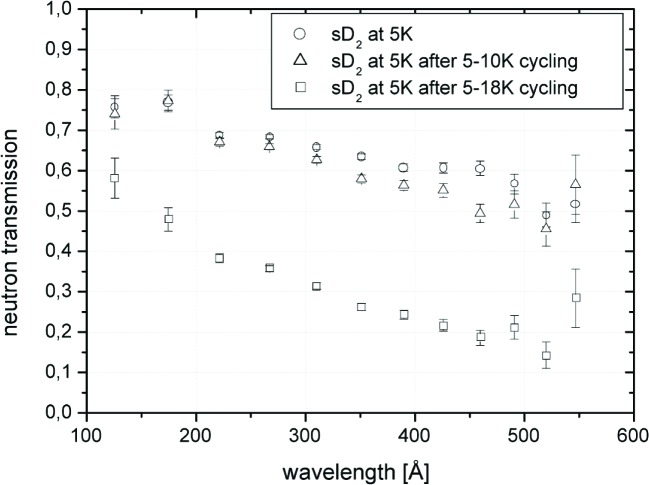
Transmission of UCN through differently treated sD_2_ samples as a function of the neutron wavelength in vacuum.

Another important reason for optical monitoring of the samples is to make sure that the neutron beam volume is filled with a known amount of material. Besides, from this information the images of sample crystals can be analyzed with respect to their light transmission. The sample is illuminated by the Raman laser from one side and photographs are taken from the opposite side. Figure 2 shows the development of the image brightness as a function of time during which the sample crystal undergoes thermal cycling. The initially high light transmission of a 5 K crystal reduces slightly during cycling the sample between 5 K and 10 K, but becomes very small after seven cycles between 5 K and 18 K. Figure 3 shows preliminary results for UCN transmission through a sD_2_ sample under the same thermal treatment as before. The initial transmission is only slightly affected by thermal cycling between 5 K and 10 K while the effect of cycling up to 18 K is dramatic.

## 4. Conclusions and Outlook

Cross sections for CN, VCN, and UCN on sD_2_ have been measured. As an example the influence of thermal cycling on the (elastic) scattering of UCN was shown. The cross sections are only slightly affected by thermal cycling between 5 K and 10 K: A pulsed UCN source can thus be operated without deterioration of the sD_2_ converter, as long as the temperature stays below 10 K, as planned for the PSI UCN source. The analysis of all the data from the experiments is under way. The experimental investigations will be extended to freezing from the gas phase, to UCN production cross section measurements on D_2_ and to a comparison with O_2_ and CD_4_ as converter materials.

## Footnotes

2 Certain commercial equipment, instruments, or materials are identified in this paper to foster understanding. Such identification does not imply recommendation or endorsement by the National Institute of Standards and Technology, nor does it imply that the materials or equipment identified are necessarily the best available for the purpose.
